# Mr. Whitford's Newly Invented Truss

**Published:** 1801-09

**Authors:** J. Whitford

**Affiliations:** Surgeons Instrument Maker. St. Bartholomew's Hospital


To the EDITORS of the MEDICAL and PHYSICAL JOURNAL.

Gentlemen,

THROUGH the medium of your very useful Publication, I beg leave to make known to the medical world, as early as possible, a newly invented Elastic Spring Truss, for the relief of those that are ruptured; and should I obtain an admission of it into youi Journal, I shall ever esteem myself,

Your's, &c.

J. WHITFORD, Surgeons Instrument Maker.

*St. Bartholomew's Hospital, Aug.* 17, 1801.

The occasional difficulties and dangers attending those persons afflicted with ruptures, for the want of a proper truss or bandage, and the bad effects which frequently occur in the application of common trusses not fitting the part affected, call loudly for some improvement. I have for several years past spent much time upon this kind of mechanism, and about three years ago I made an improvement upon the common truss, by lengthening the pad, so as to cause the spring, surrounding the body, to apply higher upon the hips of the patient, and the pad to come lower, which prevent the truss from slipping off the part, when once properly applied. This advantage has given very great satisfaction; but I thought that some improvement was still wanting, to prevent the wearing of under straps; and I have great satisfaction in informing the public, that I have at last succeeded in inventing a kind of truss, that, by the means of a spring in the pad, which makes a constant pressure, and that pressure to any degree requisite against the opening, so that the intestine is kept up, the opening in the tendon contracted, and the sides of the sac made to approach each other, so that a radical cure, in many instances, will be effected. The mechanism of the pad supersedes the necessity of under straps, which are inconvenient and galling to those who use much exercise, whereas this truss is perfectly easy to the wearer, and adapts itself to any attitude in which the body may be placed.

J. Whitford has the satisfaction of knowing, that his invention is is sanctioned by the opinion and recommendation of several of the Faculty, and by those who have most feelingly expressed their approbation, from experiencing its efficacy, in the comfort, ease, and safety obtained by it.

The inventor cannot suppress the pleasure of adding, that he is extremely happy in the reflection, that he has discovered an apparatus for the relief of a disease, which, if neglected, frequently proves the most painful and dangerous to which the human frame is liable.

